# Exploring the trade-off between quality and fairness in human partner choice

**DOI:** 10.1098/rsos.160510

**Published:** 2016-11-09

**Authors:** Nichola J. Raihani, Pat Barclay

**Affiliations:** 1 Department of Experimental Psychology, University College London, London WC1H 0AP, UK; 2 Department of Psychology, University of Guelph, Guelph, CanadaN1G 2W1

**Keywords:** partner choice, biological market theory, quality, fairness

## Abstract

Partner choice is an important force underpinning cooperation in humans and other animals. Nevertheless, the mechanisms individuals use to evaluate and discriminate among partners who vary across different dimensions are poorly understood. Generally, individuals are expected to prefer partners who are both able and willing to invest in cooperation but how do individuals prioritize the ability over willingness to invest when these characteristics are opposed to one another? We used a modified Dictator Game to tackle this question. Choosers evaluated partners varying in quality (proxied by wealth) and fairness, in conditions when wealth was relatively stable or liable to change. When both partners were equally fair (or unfair), choosers typically preferred the richer partner. Nevertheless, when asked to choose between a rich-stingy and a poor-fair partner, choosers prioritized fairness over wealth—with this preference being particularly pronounced when wealth was unstable. The implications of these findings for real-world partner choice are discussed.

## 1. Introduction

Costly helpful behaviour can evolve if individuals reap downstream benefits from their actions. One way this can be achieved is if helpful individuals are preferred as partners: giving players the option to either avoid bad partners or actively choose good ones increases cooperation, compared to interactions where individuals are forced to interact with one another [1–5]. Biological market theory [5–7] predicts that individuals prefer the ‘best’ possible partners for interactions. But what does ‘best’ actually mean? In the context of cooperation, the best partners are those who are most able *and* most willing to confer benefits on others [7–9]. In hunter–gatherer societies, for example, those who hunt successfully and are also willing to share the spoils with others form more profitable relationships than those who are successful but do not share [10]. In many cases, ability and willingness to cooperate may be correlated, but how should these qualities be traded off against one another if individuals score highly on one dimension but poorly on the other?

Here, we empirically explored how human subjects trade off ability versus willingness to cooperate when choosing a social partner; and whether these preferences are moderated by quality stability [11]. ‘Choosers’ observed the decisions made by two ‘dictators’ in a modified Dictator Game [12] and then chose which of these individuals they wanted to interact with in a second Dictator Game. Choosers evaluated dictators across three dimensions: wealth, fairness and wealth stability. We asked how choosers trade off quality against fairness when selecting a partner for cooperative interactions.

## 2. Material and methods

This project was approved by UCL Ethics Board (project 3720/001). All data were collected in April 2016 using Amazon Mechanical Turk (www.mturk.com, hereafter MTurk). MTurk is an online marketplace that connects requesters (experimenters) and workers (subjects). All individuals on MTurk participate anonymously via a unique worker ID [13]. This worker ID can also be used to prevent subjects from participating more than once in the same study [14]. The principal advantage of using MTurk to collect data is access to a more diverse subject pool than would otherwise typically be possible when recruiting subjects to a university laboratory [15]. Nevertheless, although MTurk samples are typically less biased than standard laboratory samples, it is important to note that they are still not representative of the broader population from which they are drawn [16]. Despite this caveat, studies conducted online using MTurk do not produce systematically different results from those done under more traditional laboratory or real-world settings, despite the loss of experimenter control and also the smaller stake sizes that are typically used on MTurk (e.g. [17–19]).

We recruited 788 US-based workers (375 females and 410 males; three workers did not specify; mean age: 34 ± 0.4; range: 18–73) to take part in a modified Dictator Game [12]. Of these, 298 were allocated to the role of dictator and 489 to the role of chooser. All workers were truthfully informed that the other players were real and that their identity would not be revealed to the other players in the game. Workers were also required to correctly answer two (dictators) or three (choosers) comprehension questions to be eligible to participate in the study (see electronic supplementary material, S1, for instructions).

Dictators took part in two games and were allocated to one of four conditions, which determined (i) their endowment in the first game ($2.50 or $0.50) and (ii) whether this endowment would stay the same, or be switched for the second game. Hereafter, we refer to dictators who received the larger endowment as ‘rich’ and those who receive the smaller endowment as ‘poor’ though note that these labels were not seen by subjects. In each game, dictators were informed that they could choose to send 50% or 20% of their starting endowment to another worker. For ease, these decisions are henceforth referred to as ‘fair’ and ‘stingy’, respectively. After their first decision, dictators were informed that they had been selected to play again with another worker. At this point, some of the dictators in each condition were told that their endowment for the second game was the same as in the first, while the remainder received the alternative endowment. Dictators were not aware that they were being evaluated—and chosen—on the basis of their decisions. Dictators who were not chosen for interactions still played a second game, but the amount they sent to the partner was sent to another worker from our database.

Choosers were shown the endowments and decisions made by two dictators in the first game, and then asked to select a partner for the next game, or to indicate that they had no preference (see table 1 for scenarios presented to choosers). Choosers were aware that they would be in the role of the receiver in the second game. Before deciding, choosers were informed that there was either a 10% or 50% probability that the dictators' wealth would change in the subsequent interaction, such that rich dictators would become poor and vice versa. These conditions are henceforth referred to as ‘wealth-stable’ and ‘wealth-unstable’, respectively. Choosers were finally asked to state what decision they thought most other choosers would make in that situation, in order to determine whether choosers' own preferences differed from how they thought the majority of other choosers would behave (see electronic supplementary material, table S1, for results).

**Table 1.** Choosers' partner preferences according to how the two dictators had split money in a previous game, and whether wealth was relatively stable or could change. Scenario represents the dictator decisions shown to the chooser. Rich dictators had an endowment of $2.50; poor dictators had $0.50. Fair decisions implied giving 50% of the endowment to the other worker; stingy decisions implied giving 20% to the other worker. Our primary comparison is #4 (rich-stingy versus poor-fair).

scenario	wealth (*n*)	prefer rich (%)	prefer poor (%)	no preference (%)
1. rich-fair versus poor-fair	stable (47)	78.7	8.5	12.8
	change (48)	33.3	20.8	45.8
2. rich-stingy versus poor-stingy	stable (50)	72.0	14.0	14.0
	change (49)	28.6	24.5	46.9
3. rich-fair versus poor-stingy	stable (49)	89.8	2.04	8.16
	change (48)	75.0	6.25	18.8
4. rich-stingy versus poor-fair	stable (99)	37.3	49.5	13.1
	change (99)	12.1	73.7	14.1

The experiment was designed such that rich dictators always offered higher absolute pay-offs than poor dictators, even if they were stingy. Accordingly, we predicted that (i) choosers would typically prefer rich partners, so long as wealth was stable and both partners were equally fair; but that (ii) poor-fair partners might be preferred over rich-stingy partners and that (iii) this preference would be most pronounced in the wealth-unstable condition. To test these predictions, it was not necessary to run conditions where dictators were equally wealthy, but differed in fairness. As such, we did not run conditions comparing receiver responses to rich-fair versus rich-stingy dictators, or poor-fair versus poor-stingy (see table 1 for all conditions that were run). We had no prior expectations about how accurate choosers would be when predicting the preferences of other choosers.

All data were analysed using R v. 3.0.3 (http://www.r-project.org). As we tested multiple hypotheses with the same data, we used sequential Bonferroni corrections to control the false discovery rate [20]. This method is a compromise between reducing the false discovery rate and conserving statistical power [21]. We specify *m* as the total number of statistical tests performed using data from a single table. In the results, we report uncorrected *p*-values and adjusted *α* levels. Thus, results should only be taken as conventionally significant where *p* < *α*.

## 3. Results

### 3.1. Dictator decisions

Rich and poor dictators were equally fair: analysing first decisions only, 63/148 rich dictators were fair; compared with 71/150 of the poor dictators (*χ*^2^-test: *χ*^2^ = 0.50, *p* = 0.48, *α* = 0.05). Dictator decisions were remarkably consistent: 268/298 dictators sent the same proportion of the endowment to the partner in the second game as in the first. Dictators who experienced changing wealth were slightly less consistent than those who experienced stable wealth, though this effect was not significant after controlling for multiple comparisons (*wealth-stable*: 9/140 inconsistent decisions; *wealth-unstable:* 20/129 inconsistent decisions, *χ*^2^-test: *χ*^2^ = 3.82, d.f. = 1, *p* = 0.05, *α* = 0.025).

### 3.2. Chooser decisions

**Figure 1.**
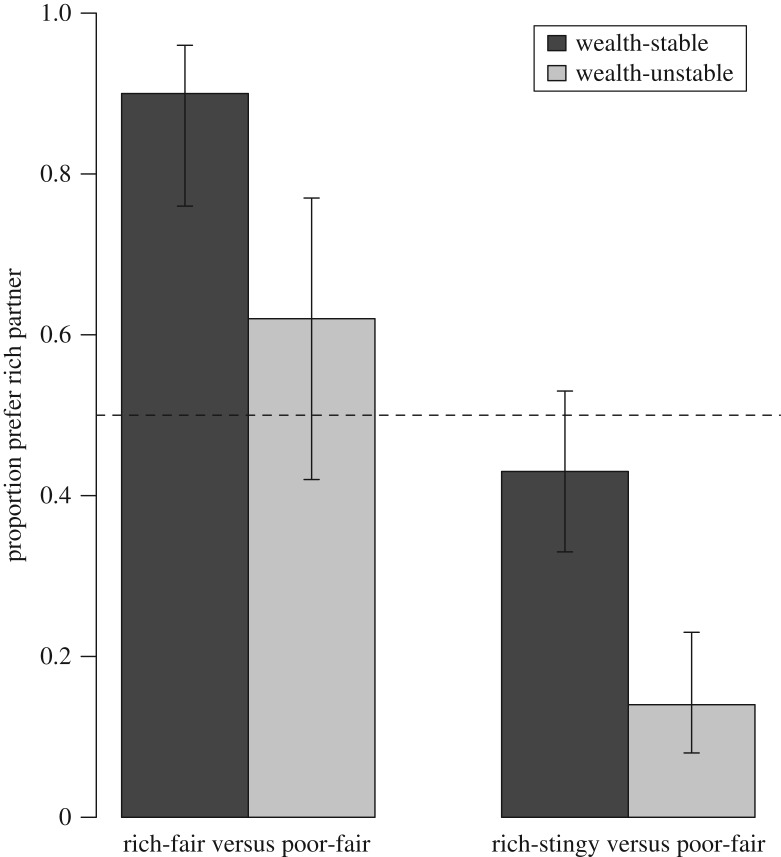
Choosers' preferences for the rich partner when choosing between rich-fair and poor-fair, and between rich-stingy and poor-fair, respectively. Dark grey bars are when wealth was stable; light grey bars are when wealth was unstable. Figure shows proportion choosers picking the rich partner (denominator is the total number of choosers who expressed a preference) with 95% binomial confidence intervals. The dotted horizontal line at 0.5 is what would be expected if choosers were selecting partners at random.

We were most interested in how choosers would evaluate dictators when wealth and fairness were opposed to one another. Under stable wealth, 49/86 (57.0%) choosers who expressed a preference chose the poor-fair partner over the rich-stingy one (table 1). Although this preference is not significantly different from chance (binomial *p* = 0.24, *α* = 0.02; figure 1), it is nevertheless notable that more than 50% of choosers preferred the poor-fair partner, when the expected pay-offs from choosing the rich partner (assuming fairness was stable) would have been larger (table 2). The preference for the poor-fair partner was enhanced in the wealth-unstable condition, where 73/85 (85.9%) choosers selected the poor-fair partner (binomial *p* < 0.001, *α* = 0.004), which is a significantly greater preference (*χ*^2^-test: *χ*^2^ = 16.1, d.f. = 1, *p* < 0.001, *α* = 0.006; figure 1). This is less surprising as the expected pay-offs of choosing the poor partner were larger than choosing the rich partner under these conditions (table 2).

**Table 2.** Expected pay-offs of choosing the rich/poor partner for the next interaction, according to whether wealth is stable or not. Note that we make the assumption that fairness is entirely consistent, such that fair players remain fair and stingy remain stingy. The last column shows the expected partner preference of an economically rational player who is maximizing expected pay-offs.

scenario	wealth	expected pay-offs (choose rich)	expected pay-offs (choose poor)	rational preference
1. rich-fair versus poor-fair	stable	$1.15	$0.35	rich
	change	$0.75	$0.75	no preference
2. rich-stingy versus poor-stingy	stable	$0.46	$0.14	rich
	change	$0.30	$0.30	no preference
3. rich-fair versus poor-stingy	stable	$1.15	$0.14	rich
	change	$0.75	$0.30	rich
4. rich-stingy versus poor-fair	stable	$0.46	$0.35	rich
	change	$0.30	$0.75	poor

Based on expected pay-offs (table 2), we predicted that participants would prefer rich partners over poor partners if both were equally fair and wealth was stable. This prediction was supported (table 1): 37/41 (90.2%) choosers preferred rich over poor dictators if both were fair (binomial *p* < 0.001, *α* = 0.005; figure 1), and 36/43 (83.7%) choosers preferred rich over poor dictators if both were stingy (binomial *p* < 0.001, *α* = 0.006; *χ*^2^-test on the difference: *χ*^2^ = 0.32, d.f. = 1, *p* = 0.57, *α* = 0.05). We also predicted that choosers should be more likely to state no preference between rich-fair/poor-fair or rich-stingy/poor-stingy partners when wealth was unstable because (assuming fairness was stable) there would be no difference in the expected pay-offs of choosing either partner (table 2). This prediction was also supported (table 1): under unstable wealth 22/48 (45.8%) choosers stated no preference when choosing between rich-fair and poor-fair partners, compared with 6/47 (12.8%) stating no preference when wealth was stable (*χ*^2^-test: *χ*^2^ = 11.0, d.f. = 1, *p* = 0.001, *α* = 0.008). Similarly, 23/49 (46.9%) choosers stated no preference when choosing between rich-stingy and poor-stingy partners under unstable wealth, compared with 7/50 (14.0%) when wealth was stable (*χ*^2^-test: *χ*^2^ = 11.2, d.f. = 1, *p* < 0.001, *α* = 0.007). As a robustness check, we asked whether choosers always preferred rich-fair over poor-stingy dictators, regardless of wealth stability. This prediction was supported: most choosers (who stated a preference) chose the rich-fair partner, regardless of whether wealth was stable (44/45, binomial *p* < 0.001, *α* = 0.004) or unstable (36/43, binomial *p* < 0.001, *α* = 0.005; Fisher's exact test on the difference: *p* = 0.33, *α* = 0.03).

## 4. Discussion

Partner choice is an important mechanism underpinning human cooperation. Many laboratory studies have demonstrated preferences for those who are willing to give, without also asking how choosers respond to differences in ability to give (e.g. [1–3], but see [10,22,23]). Incorporating such variation is crucial for understanding how partner choice mechanisms evolve [11]. Studies that have disentangled willingness from ability to give have suggested that willingness might often trump ability to give when evaluating partners [10,22,23]. For example, a recent study of cooperative hunting among members of the Australian Wester Desert Martu society found that individuals who share a higher proportion of their hunting spoils—rather than individuals who are the best hunters—enjoy more central social network positions and have access to more cooperative hunting partnerships as a consequence [23]. Similarly, under this controlled laboratory setting, we find that choosers often place a higher emphasis on fairness than wealth, even when choosing the rich partner is associated with higher expected pay-offs.

The preference for fairness is most strongly pronounced when wealth is unstable, emphasizing the important role of quality stability in mediating partner choice decisions. In this study, we exogenously varied the stability of partner quality—and choosers were aware of the relative risk that the partner's quality would change. By contrast, the stability of partner quality in real-world interactions is largely unknown. Quality is unstable if one's ability to confer benefits is largely determined by chance (as in [22]), fluctuates greatly, or does not generalize across situations. In the context of partner choice, the current findings suggest that signals of fairness will trump signals of quality as the stability of quality decreases, or as differences in quality decrease or even disappear (as in most laboratory experiments). For instance, although variation in hunting ability is likely to be a stable trait [10], many factors beyond the hunter's control also influence the probability of success on any given attempt. As such, the empirical finding that individuals attend more to cues of fairness or generosity than to skill when selecting hunting partners supports these predictions. An important future direction for research will be to quantify the stability of different aspects of partner quality in real-world interactions.

Another important consideration in future studies will be to explore how willingness and ability to give are traded off against one another when there are large disparities in quality and/or fairness in a population. For instance, it is not clear how our results might change if we assumed far larger differences, either in quality or in fairness. We suggest that the generality of the current findings could best be tackled using theoretical models, which could define the thresholds at which individuals are willing to accept high-quality, unfair partners over lower quality, fair ones. Similar approaches have been used in the context of understanding mate choice in females—specifically for defining the thresholds at which females should accept mating polygynously with a high-quality male versus mating monogamously with an inferior male [24]—and could be used to understand partner choice in other contexts.

In this study, the fact that choosers preferred poor-fair partners over rich-stingy ones (particularly when wealth was unstable) indicates that they believed that fairness was a relatively stable trait. How justified is this belief? Theoretical models support the idea that costly cooperation can be a consistent trait: initial variation in a population (arising through mutation or other random processes) can favour the evolution of responsive individuals, who choose or avoid partners on the basis of their actions [25]. These responsive individuals can, in turn, favour the evolution of consistency in social behaviours, even when these behaviours are costly to perform [25–27]. The assumption that cooperativeness is a relatively stable trait also has empirical support: social decision-making is relatively stable over time and contexts [28], even in relatively ‘noisy’ environments like MTurk [19]. Most importantly, in the context of the current study, dictator decisions in game 1 were highly predictive of their decisions in game 2, even (albeit to a lesser extent) when their wealth changed. Thus, choosers could reliably infer dictators' future behaviour from their previous actions.

Although fairness in game 1 was predictive of fairness in game 2, it is somewhat surprising that over 50% of choosers stated an (economically irrational) preference for a poor-fair partner (over a rich-stingy one) when wealth was stable. Under the assumption that fairness is a stable trait, the expected pay-offs associated with choosing the rich-stingy partner outweigh those associated with choosing the poor-fair one when wealth is stable (table 2), suggesting that choosers are often willing to sacrifice pay-offs in order to select a fair partner. One simple explanation for this could be that choosers simply miscalculated the pay-offs associated with choosing the rich or poor partner, respectively. Though possible, this explanation seems relatively unlikely as chooser preferences (or lack of preference) aligned with expected pay-offs in all other conditions. Instead, we suggest that the avoidance of rich-stingy dictators can also be interpreted as a form of third-party punishment [29]: by incurring a cost to choose the poor-fair partner, choosers may have believed that they were preventing the rich-stingy dictator from taking part in another game and thereby imposing a punishment cost on this individual. Indeed, choosers were not informed that dictators would play with another individual, even if the chooser did not choose them. Proximate motives to invest in third-party punishment can be supported on a functional level if, for example, the punisher derives signalling benefits from their actions [30,31]. To explore this idea further, in future studies, it would be interesting to determine whether individuals who forgo expected pay-offs to reject rich-stingy partners are also more likely to invest in conventional third-party punishment of a stingy dictator, and whether they gain reputation benefits for their actions (e.g. [31,32]). Although several studies have shown how rejecting an uncooperative partner can be a self-serving decision [33], no study to our knowledge has described scenarios where partner rejection could operate as a form of costly third-party punishment (i.e. when accepting an uncooperative partner would nevertheless yield higher pay-offs than accepting a cooperative one). This suggests interesting avenues for further theoretical and empirical exploration.

## Supplementary Material

SI: game instructions; additional detail on analysis

## Supplementary Material

SI_data: raw data file

## Data accessibility

All data necessary to reproduce analyses have been uploaded to Figshare (https://dx.doi.org/10.6084/m9.figshare.3997200).

## References

[1] Barclay P, Willer R 2007 Partner choice creates competitive altruism in humans. Proc. R. Soc. B 274, 749–753. (doi:10.1098/rspb.2006.0209) 10.1098/rspb.2006.0209 PMC2197220 17255001

[2] Sylwester K, Roberts G 2010 Cooperators benefit through reputation-based partner choice in economic games. Biol. Lett. 6, 659–662. (doi:10.1098/rsbl.2010.0209) 20410026 10.1098/rsbl.2010.0209 PMC2936156

[3] Sylwester K, Roberts G 2013 Reputation-based partner choice is an effective alternative to indirect reciprocity in solving social dilemmas. Evol. Hum. Behav. 34, 201–206. (doi:10.1016/j.evolhumbehav.2012.11.009)

[4] Bshary R, Grutter AS 2002 Experimental evidence that partner choice is a driving force in the payoff distribution among cooperators or mutualists: the cleaner fish case. Ecol. Lett. 5, 130–136. (doi:10.1046/j.1461-0248.2002.00295.x)

[5] Barclay P 2016 Biological markets and the effects of partner choice on cooperation and friendship. Curr. Opin. Psychol. 7, 33–38. (doi:10.1016/j.copsyc.2015.07.012)

[6] Noë R, Hammerstein P 1995 Biological markets. Trends Ecol. Evol. 10, 336–339. (doi:10.1016/S0169-5347(00)89123-5) 21237061 10.1016/s0169-5347(00)89123-5

[7] Barclay P 2013 Strategies for cooperation in biological markets, especially for humans. Evol. Hum. Behav. 34, 164–175. (doi:10.1016/j.evolhumbehav.2013.02.002)

[8] Andre JB, Baumard N 2011 The evolution of fairness in a biological market. Evolution 65, 1447–1456. (doi:10.1111/j.1558-5646.2011.01232.x) 21521194 10.1111/j.1558-5646.2011.01232.x

[9] Baumard N, Andre JB, Sperber D 2013 A mutualistic approach to morality: the evolution of fairness by partner choice. Behav. Brain Sci. 36, 59–78. (doi:10.1017/S0140525X11002202) 23445574 10.1017/S0140525X11002202

[10] Gurven M, Allen-Arave W, Hill KR, Hurtado M 2000 ‘It's a wonderful life’: signaling generosity among the Ache of Paraguay. Evol. Hum. Behav. 21, 263–282. (doi:10.1016/S1090-5138(00)00032-5) 10899478 10.1016/s1090-5138(00)00032-5

[11] McNamara JM, Leimar O 2010 Variation and the response to variation as a basis for successful cooperation. Proc. R. Soc. B 365, 2627–2633. (doi:10.1098/rstb.2010.0159) 10.1098/rstb.2010.0159 PMC2936179 20679107

[12] Kahneman D, Knetsch JL, Thaler R 1986 Fairness as a constraint on profit seeking: entitlements in the market. Am. Econ. Rev. 76, 728–741.

[13] Mason W, Suri S 2012 Conducting behavioral research on Amazon's Mechanical Turk. Behav. Res. Meth. 44, 1–23. (doi:10.3758/s13428-011-0124-6) 10.3758/s13428-011-0124-6 21717266

[14] Rand DG 2012 The promise of Mechanical Turk: How online labor markets can help theorists run behavioral experiments. J. Theor. Biol. 299, 172–179. (doi:10.1016/j.jtbi.2011.03.004) 21402081 10.1016/j.jtbi.2011.03.004

[15] Buhrmester M, Kwang T, Gosling SD 2011 Amazon's Mechanical Turk: a new source of inexpensive, yet high-quality, data? Persp. Psychol. Sci. 6, 3–5. (doi:10.1177/1745691610393980) 10.1177/1745691610393980 26162106

[16] Paolacci G, Chandler J 2014 Inside the Turk. Curr. Dir. Psychol. Sci. 23, 184 (doi:10.1177/0963721414531598)

[17] Horton JJ, Rand DG, Zeckhauser RJ 2011 The online laboratory: conducting experiments in a real labor market. Exp. Econ. 14, 399–425. (doi:10.1007/s10683-011-9273-9)

[18] Raihani NJ, Mace R, Lamba S 2013 The effect of $1, $5 and $10 stakes in an online Dictator Game. PLoS ONE 8, e73131 (doi:10.1371/journal.pone.0073131) 23951342 10.1371/journal.pone.0073131 PMC3741194

[19] Peysakhovich A, Nowak MA, Rand DG 2014 Humans display a ‘cooperative phenotype’ that is domain general and temporally stable. Nat. Comm. 5, 4939 (doi:10.1038/ncomms5939) 10.1038/ncomms5939 25225950

[20] Benjamini Y, Hochberg Y 1995 Controlling the false discovery rate: a practical and powerful approach to multiple testing. J. R. Stat. Soc. B, 57, 289–300.

[21] Waite TA, Campbell LG 2006 Controlling the false discovery rate and increasing statistical power in ecological studies. Ecoscience 13, 439–442. (doi:10.2980/1195-6860(2006)13[439:CTFDRA]2.0.CO;2)

[22] Delton AW, Robertson TE 2012 The social cognition of social foraging: partner selection by underlying valuation. Evol. Hum. Behav. 33, 715–725. (doi:10.1016/j.evolhumbehav.2012.05.007) 23162372 10.1016/j.evolhumbehav.2012.05.007 PMC3498050

[23] Bird RB, Power EA 2015 Prosocial signaling and cooperation among Martu hunters. Evol. Hum. Behav. 36, 389–397. (doi:10.1016/j.evolhumbehav.2015.02.003)

[24] Orians GH 1969 On the evolution of mating systems in birds and mammals. Am. Nat. 103, 589–603. (doi:10.1086/282628)

[25] McNamara JM, Barta Z, Fromhage L, Houston AI 2008 The coevolution of choosiness and cooperation. Nature 451, 189–192. (doi:10.1038/nature06455) 18185587 10.1038/nature06455

[26] McNamara JM, Houston AI 2002 Credible threats and promises. Phil. Trans. R. Soc. Lond. B 357, 1607–1616. (doi:10.1098/rstb.2002.1069) 12495517 10.1098/rstb.2002.1069 PMC1693069

[27] Wolf M, Van Doorn GS, Weissing FJ 2011 On the coevolution of social responsiveness and behavioural consistency. Proc. R. Soc. B 278, 440–448. (doi:10.1098/rspb.2010.1051) 10.1098/rspb.2010.1051 PMC3013403 20739321

[28] Kurzban R, Houser D 2005 Experiments investigating cooperative types in humans: a complement to evolutionary theory and simulations. Proc. Natl Acad. Sci. USA 102, 1803–1807. (doi:10.1073/pnas.0408759102) 15665099 10.1073/pnas.0408759102 PMC547861

[29] Fehr E, Fischbacher U 2004 Third-party punishment and social norms. Evol. Hum. Behav. 25, 63–87. (doi:10.1016/S1090-5138(04)00005-4)

[30] Raihani NJ, Bshary R 2015 The reputation of punishers. Trends Ecol. Evol. 30, 98–103. (doi:10.1016/j.tree.2014.12.003) 25577128 10.1016/j.tree.2014.12.003

[31] Barclay P 2006 Reputational benefits for altruistic punishment. Evol. Hum. Behav. 27, 325–344. (doi:10.1016/j.evolhumbehav.2006.01.003)

[32] Raihani NJ, Bshary R 2015 Third-party punishers are rewarded, but third-party helpers even more so. Evolution 69, 993–1003. (doi:10.1111/evo.12637) 25756463 10.1111/evo.12637

[33] Raihani NJ, Thornton A, Bshary R 2012 Punishment and cooperation in nature. Trends Ecol. Evol. 27, 288–295. (doi:10.1016/j.tree.2011.12.004) 22284810 10.1016/j.tree.2011.12.004

