# Supplementary material for: Exploring the trade-off between quality and fairness in human partner choice
Source: R Soc Open Sci. 2016 Nov 9;3(11):160510. doi: 10.1098/rsos.160510 (PMC5180136; doi:10.1098/rsos.160510)
Supplement: SI: game instructions; additional detail on analysis [file rsos160510supp1.pdf]

Game Instructions (seen by chooser) from Exploring the trade-off between quality and fairness in human partner choice

You are about to take part in an academic study which is run by the Raihani Lab, based at University College London. By continuing with the HIT you are consenting to allow the Raihani Lab to use your responses in the study for academic purposes.

We will ask you some questions about yourself and your household, and also ask you to play a brief game with another Mturk worker. All data are anonymous (your name or worker ID will not appear in any publication related to this study and will not be shared with any other parties).

****

Please tick 'I agree' if you agree to these conditions. If you do not wish to participate, or if you change your mind during the course of the study, please return to the Mechanical Turk Interface and click 'Return HIT'.

****

Please enter your Mechanical Turk Worker ID. This is needed to ensure you get your bonus.

It is easier for us if you do this by copying and pasting your ID (by opening the link below in a new window)

https://www.mturk.com/mturk/dashboard

****

We'd like to ask you two questions about yourself.

1. Please tell us your gender (M/F/Prefer not to disclose)
2. Please enter your age in years.

****

Thanks for telling us about yourself.

You will play a game with another MTurk worker. You will not find out the other worker's ID and they will not find out yours.

Your role in the game is to choose whether you would like worker 1 or worker 2 to be your partner.

First, you will be shown the decisions made by worker 1 and worker 2 in another game. Both workers were given a bonus and asked to **decide whether to send 50 % or 20 % of the bonus** to another worker (worker 3).

These are the bonuses worker 1 and worker 2 got in the previous game and the decisions they made.

In this case, both workers gave 50 % of their starting bonus to worker 3.

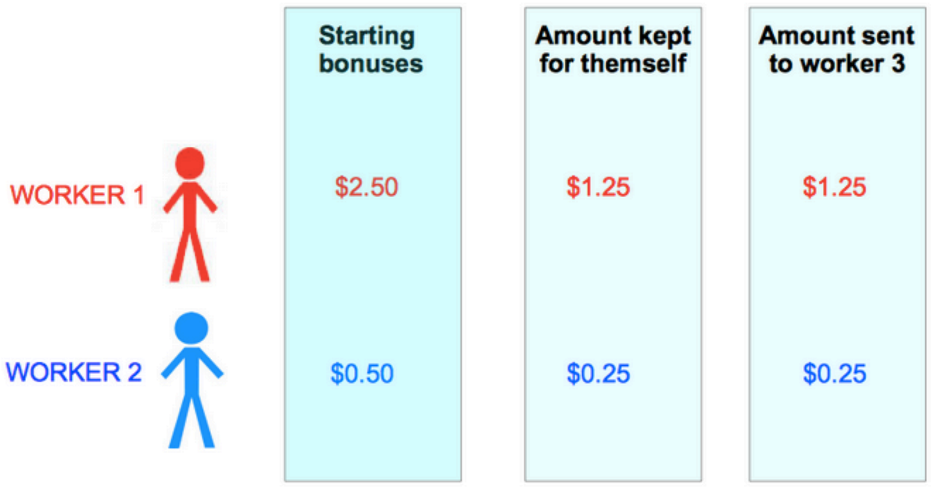

****

In a moment, you will choose whether you would like to have worker 1 or worker 2 as your partner for the same sort of game.

The worker you choose as your partner will receive another starting bonus and will be asked to decide between sending 50 % or sending 20 % of that bonus to you.

Before you choose, you should know that there is a **50 % chance** that the starting bonus of the workers will **change**, so that worker 1 will start with $0.50 and worker 2 will start with $2.50.

There is also a **50 % chance** that the starting bonuses of the workers will **remain the same**, so that worker 1 will start with $2.50 and worker 2 will start with $0.50.

Your bonus will depend on the starting bonus your partner receives AND the decision they make.

****

Before you decide, please answer the following comprehension questions:

1. Did worker 1 or worker 2 give a larger absolute amount to worker 3?

2. What is the chance that worker 1 and worker 2 will have the same starting bonus if you choose them as your partner for the next game?

3. Who gave a larger PERCENTAGE of their bonus to worker 3?

****

Well, done you answered the comprehension questions correctly!

Now, please select the worker you would like as your partner for the next round. You will receive a bonus depending on the starting bonus received by your partner, as well as the amount they decide to transfer to you.

- I would like Worker 1 to be my partner
- I would like Worker 2 to be my partner
- I don't have a preference; allocate me a partner at random

Out of interest, which worker do you think most other people would choose?

(feel free to explain your choice in the box provided)

- Most people would choose Worker 1
- Most people would choose Worker 2
- Most people would not care who they got partnered with
- I have no idea
