# Supplementary material for: Exploring the trade-off between quality and fairness in human partner choice
Source: R Soc Open Sci. 2016 Nov 9;3(11):160510. doi: 10.1098/rsos.160510 (PMC5180136; doi:10.1098/rsos.160510)
Supplement: SI_data: raw data file [file rsos160510supp2.pdf]

Table S1. Chooser predictions about others' preferences. From 'Exploring the trade-off between quality and fairness in human partner choice'.

Scenario	Wealth (N)	Predict other prefer rich	Predict others prefer poor	Predict others show no preference	Unsure who others would prefer
1. Rich-Fair vs Poor-Fair	Stable (47)	39	4	3	1
	Change (48)	22	4	14	8
2. Rich-Stingy vs Poor-Stingy	Stable (50)	43	5	0	2
	Change (49)	28	6	10	5
3. Rich-Fair vs Poor-Stingy	Stable (49)	48	1	0	0
	Change (48)	47	1	0	0
4. Rich-Stingy vs Poor-Fair	Stable (99)	53	32	3	11
	Change (99)	20	66	5	8

We asked whether chooser's preferences aligned with how they thought others would behave, specifically when choosing between rich-stingy and poor-fair partners. Indeed, choosers' own preferences did not always align with what they thought the normative response was (Table S1). Under unstable wealth, 12/85 (14.1 %) choosers preferred the rich-stingy partner, and 20/86 (23.2 %) thought others would make the same choice (Chi-squared test: $\chi^2 = 1.78$, df = 1, p = 0.18, $\alpha = 0.01$). However, when wealth was stable, 37/86 (43.0 %) choosers preferred the rich-stingy partner but thought 53/85 (62.4 %) others would have this preference (Chi-squared test: $\chi^2 = 5.65$, df = 1, p = 0.02, $\alpha = 0.01$). Note that this discrepancy between choosers' own preferences and how they thought others would behave was not significant under the corrected α level.
